# Effect of *Hantavirus* Infection on the Rodent Lung Microbiome: Specific Regulatory Roles of Host Species and Virus Types

**DOI:** 10.3390/microorganisms14010244

**Published:** 2026-01-21

**Authors:** Yaru Xiong, Zhihui Dai, Fangling He, Rongjiao Liu, Juan Wang, Zhifei Zhan, Huayun Jia, Shengbao Chen, Liang Cai

**Affiliations:** Hunan Provincial Center for Disease Control and Prevention (Hunan Academy of Preventive Medicine), Hunan Provincial Key Laboratory of Microbial Molecular Biology, Changsha 410005, China; 18373139086@163.com (Y.X.);

**Keywords:** *Hantavirus*, zoonosis, microbiome, biomarker, metagenomics

## Abstract

The lung-targeting characteristic of *Hantavirus* infection and the unclear mechanism underlying its interaction with the lung microbiome hampers the development of effective prevention and control strategies. In this study, lung tissues from *Apodemus agrarius* and *Rattus norvegicus* were collected at *Hantavirus* surveillance sites in Hunan Province. Metagenomic sequencing was subsequently applied to compare microbiome diversity, community structure, and function between infected and uninfected groups. Then the linear discriminant analysis effect size (LEfSe) was employed to identify key biomarkers. The results indicated that after infection with *Hantaan virus* (HTNV), *Apodemus agrarius* exhibited significantly increased evenness but markedly decreased richness of lung microbial communities, as reflected by consistent reductions in the number of observed species, Abundance-based Coverage Estimator (ACE) index, and Chao1 index. In contrast, *Rattus norvegicus* infected with *Seoul virus* (SEOV) showed no significant difference in microbial richness compared with uninfected controls, and even a slight increase was observed. These findings suggest that host species and virus type may play an important role in shaping microbial community responses. Furthermore, β-diversity analysis showed that the community structure was clearly separated by the host rodent species, as well as by their virus infection status. LEfSe analysis identified taxa with discriminatory power associated with infection status. *Streptococcus agalactiae* and *Streptococcus* were associated with SEOV-infected *Rattus norvegicus*, while *Chlamydia* and *Chlamydia abortus* were relatively enriched in uninfected *Apodemus agrarius*. This exploratory study reveals preliminary association between specific host—*Hantavirus* pairings (HTNV—*Apodemus agrarius* and SEOV—*Rattus norvegicus*) and the rodent lung microbiome, offering potential insights for future research into viral pathogenesis.

## 1. Introduction

*Hantavirus*, an important member of the *Bunyaviridae* family, is a globally concerned zoonotic pathogen that can cause hemorrhagic fever with renal syndrome (HFRS) [1]. Globally, *Hantavirus* outbreaks and zoonotic transmission remain active, as reflected in recent surveillance and pathogenicity studies [2]. In China, reported cases of HFRS account for 70% to 90% of the global total, and its epidemic range covers most provinces and regions across the country [3]. Existing studies have confirmed that *Hantavirus* causes disease primarily by directly damaging vascular endothelial cells and inducing immunopathological reactions. As one of its core target organs, the lung often develops typical lesions such as alveolar septal inflammation and pulmonary edema after infection [2,4]. However, current research has primarily focused on viral genomic characteristics, virus–host immune interactions, and other dimensions, with insufficient attention to host microecosystems, particularly changes in the lung tissue microbiome during infection [5,6]. This knowledge gap represents a critical obstacle in the in-depth elucidation of the pathogenic mechanism of *Hantavirus*.

The onset and progression of respiratory diseases are often not the result of the independent action of pathogens but rather a reflection of their complex interactions with the host microbiome, a hidden organ [7]. The lung tissue microbiome, a complex community colonizing the respiratory mucosa, plays an irreplaceable role in maintaining local pulmonary immune balance and resisting pathogen invasion, with its homeostasis in diversity and abundance being crucial [7,8]. Compared to the extensively studied gut microbiome, the lung microbiome exists in a markedly different physiological environment characterized by lower biomass, higher oxygen availability, and direct exposure to inhaled substances. Such interactions have been demonstrated in various infectious diseases. For instance, SARS-CoV-2 infection can induce bidirectional migration of the respiratory and gut microbiome in severe cases, and the enrichment level of specific microbiota is directly associated with disease severity [9]. *Aspergillus* infection creates a microaerophilic environment by consuming oxygen in the lungs, markedly remodels the composition of the lung microbiome, and such changes exert a feedback effect on the infection process [10]. Based on this, it is reasonable to hypothesize that in *Hantavirus*-infected rodent models, the lung tissue microbiome may also undergo similar remodeling. Virus-induced pulmonary inflammation and tissue damage may disrupt the original microbial homeostasis. Conversely, reduced microbial diversity or altered abundance of specific taxa may also inversely affect viral replication and pathogenic processes by regulating host immune responses.

Using *Hantavirus*-infected rodents as research subjects represents an ideal approach to precisely address the aforementioned scientific questions. As the natural reservoir hosts of *Hantavirus*, rodents exhibit key commonalities with human infections, such as the absence of significant clinical symptoms coupled with persistent infection and viral shedding [11]. This offers a natural model to study how long-term viral colonization affects the host lung microbiome [12]. Additionally, the use of rodent models helps control confounding factors such as genetic background and environmental exposure, thereby enabling precise assessment of the relationships among lung microbiome diversity and abundance, viral replication kinetics, and host immune responses. In recent years, novel *Hantaviruses* have been identified in North American prairie voles, with infection rates in host populations stabilizing at around 20% [13], which further supports establishing a lung tissue—focused *Hantavirus*-microbiome research system.

Importantly, recent studies indicate that land use changes driven by human activities significantly influence *Hantavirus* transmission dynamics [13,14]. For example, analysis of 42 years of monitoring data from Huxian County, Shaanxi Province, revealed that large—scale land consolidation led to a 53% decline in rodent diversity. This shifted the rodent community from one dominated by multiple species to dominance by a single species, *Apodemus agrarius*, substantially increasing *Hantavirus* transmission risk [15]. Such ecological shifts may further alter the microbiome composition of individual rodents, potentially changing virus—host interaction patterns. Similarly, studies in Madagascar have demonstrated that forest conversion to agricultural land raises *Hantavirus* transmission risk by modifying rodent habitats [16]. These macroecological studies provide essential context for microscale investigations into *Hantavirus*-lung microbiome interactions.

This study aimed to identify key microbial taxa potentially associated with *Hantavirus* infection by comparing the lung microbiota between infected and uninfected rodents. This work will help clarify the interaction mechanisms between *Hantavirus* and the microbiome, potentially revealing new pathogenic pathways and filling the research gap in viral infection-host microecology interactions. Moreover, the key microbial markers identified may serve as bioindicators in future studies for predicting disease outcomes and assessing zoonotic transmission risk, supporting early warning systems for *Hantavirus* diseases. The corresponding findings will provide scientific support for developing prevention and control strategies based on microecological regulation in rodent hosts, thereby reducing the risk of cross-species virus transmission. These outcomes hold significant theoretical guidance and practical value for the source-directed prevention and control of *Hantavirus* diseases.

## 2. Materials and Methods

### 2.1. Sample Collection and Experimental Design

Rodents were captured monthly from January to December 2024 using overnight traps set in a 5 m  ×  5 m grid at HFRS surveillance sites in Hunan Province, continuous annual sampling and parallel experimental were designed to mitigate potential seasonal influences. The surveillance sites were situated in rural and peri-urban areas of Hunan Province, these locations typically exhibited a mosaic of land use types, including small—scale agricultural fields, adjacent woodland or shrubland patches, and nearby human dwellings. Species were initially identified in the field based on morphological characteristics. Subsequently, the rodents were dissected in a dedicated sterile biological safety cabinet. During dissection, lung tissues were aseptically collected using sterilized instruments to minimize exogenous contamination. The collected tissue samples were immediately preserved in 95% ethanol and stored at −80 °C prior to use. All samples were initially screened for *Hantavirus* using RT-PCR, with a Ct value of less than 28 defined as positive. Based on the initial screening results, this study ultimately included *Hantavirus*-positive samples (n = 8) and *Hantavirus*-negative control samples (n = 6) for subsequent microbial community analysis.

The samples were grouped as follows: Group A included *Hantaan virus*-positive samples from *Apodemus agrarius* (n = 6); Group C included *Hantaan virus*-negative samples from *Apodemus agrarius* (n = 3); Group B included *Seoul virus*-positive samples from *Rattus norvegicus* (n = 2); Group D included *Seoul virus*-negative samples from *Rattus norvegicus* (n = 3). This grouping design allowed us to simultaneously assess the effects of host species (*Apodemus agrarius* vs. *Rattus norvegicus*) and viral infection status (positive vs. negative) on the lung microbiome, as well as potential interaction effects between these two factors.

### 2.2. DNA Extraction and Metagenomic Sequencing

The extraction of microbial genomic DNA from all rodent lung tissues was performed using the DNeasy PowerSoil Pro Kit (QIAGEN GmbH, Hilden, Germany; catalog no. 47014), in accordance with the standard protocol of manufacturer. Subsequently, sequencing analysis was commissioned to Sangon Biotech Co., Ltd. (Shanghai, China), which constructed shotgun metagenomic sequencing libraries and performed PE150 paired-end sequencing on the DNBSEQ-T7 ultra-high-throughput sequencing platform. The DNBSEQ^TM^ technology employed by this platform can effectively reduce sequencing error rates and index hopping, thus ensuring data quality. The average sequencing depth was 10 Gb per sample.

### 2.3. Contamination Control and Negative Controls

Given that lung tissue is a low-biomass sample source, stringent measures were implemented to assess and mitigate potential contamination introduced during DNA extraction and library preparation. Extraction blank controls: for each batch of DNA extraction, a blank control was processed in parallel. This control contained all extraction reagents and underwent identical procedures and environmental exposure as the experimental samples, but without the addition of any lung tissue, to monitor potential exogenous nucleic acid contamination derived from reagents, consumables, or the laboratory environment. Screening of negative controls via qPCR: as metagenomic sequencing was not performed on these extraction blanks, quantitative PCR (qPCR) was employed to evaluate the presence of bacterial and fungal DNA contaminants. Although extraction blanks processed in parallel showed no significant qPCR signal, indicating the absence of gross contamination, they were not sequenced due to budget constraints and the initial study design’s focus on sample comparison. The lack of sequenced library controls limits bioinformatic identification and removal of potential low-level contaminant sequences. Therefore, interpretations—particularly of low-abundance taxa—should be made cautiously. Future studies are strongly recommended to include sequenced negative controls to enable effective bioinformatic decontamination.

For bacterial contamination assessment, universal bacterial 16S rRNA gene primers were used:

27F (5′-AGAGTTTGATCMTGGCTCAG-3′)

1492R (5′-TACGGYTACCTTGTTACGACTT-3′)

For fungal contamination assessment, universal fungal ITS region primers were used:

ITS1 (5′-AACTTAAAGGAATTGACGGAAG-3′)

ITS4 (5′-GCATCACAGACCTGTTATTGCCTC-3′)

### 2.4. Bioinformatics Analysis

#### 2.4.1. Quality Control of Sequencing Data

Raw sequencing data were subjected to quality control using Fastp (v0.23.0), which included removing low-quality bases (quality score < 20), adapter sequences, and reads shorter than 50 bp. To obtain pure microbial data, the quality-controlled data were aligned to the host reference genomes (genomes of *Apodemus agrarius* and *Rattus norvegicus*) using Bowtie2 (v2.4.5), and all aligned sequences were removed. The resulting high-quality data were used for downstream analyses. High-quality microbial data were retained after quality control and host contamination removal. The raw metagenomic sequencing data generated in this study have been deposited in the NCBI Sequence Read Archive (SRA) under accession number PRJNA1378084 (Submission ID: SUB15818421).

#### 2.4.2. Metagenomic Assembly, Binning, and Gene Catalog Construction

To obtain a comprehensive set of microbial genomic information, a two-step hybrid assembly strategy was employed. First, MEGAHIT (v1.2.9) was used for co-assembly of quality-controlled reads from all samples. Subsequently, Bowtie2 (v2.4.5) was used to map reads from each sample back to the initial assembly. The unmapped reads were extracted and assembled using SPAdes (v3.15.5) in metagenomic mode to recover genomic content from low-abundance microorganisms. The contigs from both assembly steps were combined to form a comprehensive, non-redundant contig set.

Metagenomic binning was performed to reconstruct Metagenome-Assembled Genomes (MAGs). The integrated contigs were processed using the MetaWRAP pipeline (https://github.com/bxlab/metaWRAP, accessed on 15 January 2026), which leverages sequence composition (tetranucleotide frequency) and abundance profiles across samples to cluster contigs into bins. These bins were subsequently refined, quantified, reassembled, and taxonomically profiled to generate MAGs with high completeness and low contamination.

Open reading frames (ORFs) were predicted from the final contig set using Prodigal (v2.6.3) in metagenomic mode. Predicted genes with a length of ≥100 bp were retained and translated into amino acid sequences. A non-redundant gene catalog was constructed by clustering all predicted protein sequences at 95% identity using CD-HIT (v4.8.1).

#### 2.4.3. Gene Abundance Quantification and Annotation

The abundance of each gene in the non-redundant catalog was quantified per sample. Clean reads from each sample were aligned to the gene catalog using Bowtie2 (v2.4.5). The number of mapped reads per gene was counted, normalized by gene length, and used to represent gene abundance.

For functional and taxonomic annotation, the protein sequences of the non-redundant gene catalog were aligned against several public databases using BLASTP (https://blast.ncbi.nlm.nih.gov/Blast.cgi, accessed on 15 January 2026), including the NCBI NR database (for taxonomy), KEGG, eggNOG, ARDB, CAZy, and SEED. The highest-scoring hit meeting defined thresholds (e-value < 1e-5, identity > 30%, coverage > 70%) was assigned to each gene. The resulting annotations, combined with the gene abundance profiles, were used to generate taxonomic and functional abundance tables for downstream statistical and comparative analyses.

### 2.5. Statistical Analysis

#### 2.5.1. Analysis of Microbial Community Diversity

Circos plots were used in taxonomic analysis to illustrate species clustering and relative abundance in each sample, compare differences among different samples, and visualize the composition of bacterial communities at different taxonomic levels across samples.

In α-diversity analysis, microbial diversity and richness were evaluated using the Shannon index, Simpson index, observed species number, ACE, and Chao1 index. Differences between groups were examined with the Kruskal–Wallis test after confirming that the data did not follow a normal distribution.

β-diversity employed principal coordinate analysis (PCoA) and non-metric multidimensional scaling (NMDS) to reflect the similarity and dissimilarity among communities. Calculations were performed using the Bray–Curtis distance matrix. Differences in community structure between groups were assessed using Permutational Multivariate Analysis of Variance (PERMANOVA, implemented with the adonis2 function) and Analysis of Similarities (ANOSIM). Both tests were based on the Bray–Curtis distance matrix with 999 permutations. The PERMANOVA model included host species, infection status, and their interaction term as the primary factors of interest.

#### 2.5.2. Screening for Intergroup Differential Species

The LEfSe analysis was performed to identify differentially abundant taxonomic groups (from phylum to genus) and KEGG pathways across experimental groups. Prior to this analysis, the abundance of each KEGG pathway in a sample was calculated as the sum of the abundances of all genes annotated to that pathway via KEGG Orthology (KO) identifiers. The analysis itself employed a multi-step statistical approach: significant differences in feature abundance were first identified using the Kruskal–Wallis test (*p* < 0.05, with the False Discovery Rate (FDR) applied for multiple-testing correction), followed by pairwise testing with the Wilcoxon rank-sum test to ensure biological consistency. Finally, features that remained significant were assessed using Linear Discriminant Analysis (LDA) to estimate their effect size, and only those with an LDA score > 3.5 were considered robust biomarkers.

#### 2.5.3. Control of Covariates and Confounding Factors

To account for potential confounding factors such as sampling season and capture location, these variables were included as covariates in the PERMANOVA model. Although detailed habitat classification (forest vs. agricultural land) was not recorded for each individual trap, the “capture location” covariate accounted for broad spatial variation across the surveillance sites. For continuous variables (such as α-diversity indices), nonparametric tests were used if they did not follow a normal distribution. All statistical analyses were performed in the R environment (v4.2.0), and data visualization was conducted using the ggplot2 package (https://ggplot2.tidyverse.org/, accessed on 15 January 2026).

### 2.6. Sample Size Consideration

The sample sizes in this exploratory study (n = 6 for group A, n = 3 for group C, n = 2 for group B, and n = 3 for group D) were primarily determined by the field availability of PCR—confirmed, species- and virus type-matched specimens from ongoing *Hantavirus* surveillance. While this limited n precluded a formal a priori power calculation, we adopted a rigorous metagenomic sequencing depth (average 10 Gb per sample) and implemented stringent bioinformatic and statistical controls (contamination assessment, PERMANOVA with covariates, non-parametric tests, and FDR correction in LEfSe) to maximize the robustness of the observations within this constraint. It is acknowledged that statistical power and generalizability are limited by the small and uneven group sizes, particularly for Group B (n = 2). Therefore, all results and interpretations are framed as preliminary and hypothesis-generating, with validation required in larger controlled cohorts.

## 3. Results

### 3.1. Assessment of Procedural Contamination and Microbial Composition

To ensure the reliability of the low-biomass lung microbiome data, potential procedural contamination was first assessed. Extraction blank controls, which were processed alongside tissue samples but without biological material, were screened via quantitative PCR (qPCR) using universal bacterial and fungal primer sets (Table 1). No detectable amplification, i.e., cycle threshold (Ct) values > 35, was observed in any blank control, indicating that contaminating nucleic acids from reagents or the extraction process were below the detection limits of the qPCR assays used. However, the absence of sequenced library controls limits the ability to bioinformatically identify and subtract potential contaminant sequences present at low levels.

**Figure 1:**
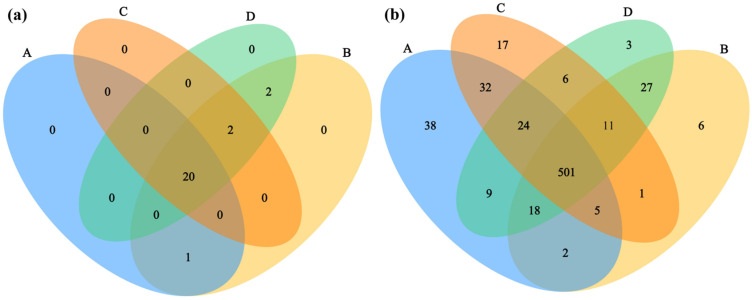
Venn diagrams comparing the taxonomic composition shared between different sample groups at the (**a**) phylum and (**b**) genus levels. Sample sizes per group were as follows: A (n = 6), B (n = 2), C (n = 3), and D (n = 3).

Through metagenomic sequencing analysis of different rodent lung tissue samples, the characteristics of the microbial composition and structure were systematically evaluated at the phylum and genus levels. Venn diagram analysis (Figure 1) revealed that, at the phylum level (Figure 1a), no unique microbial taxa were detected exclusively in either the *Hantaan virus*-positive (Group A) or virus-negative (Group C) samples of *Apodemus agrarius*. Two microbial taxa were shared specifically between the *Seoul virus*-positive (Group B) and virus-negative (Group D) samples of *Rattus norvegicus*, while 20 microbial taxa were common across all four groups. At the genus level (Figure 1b), Group A contained 38 unique genera, Group C contained 17, Group D contained 3, and Group B contained 6. A total of 501 genera were shared between all groups—a substantially higher number than that observed at the phylum level. These results suggest that the influence of specific host–virus pairings on the lung microbiota is more pronounced at the genus level. The effects of the host species and virus type were partially confounded, as HTNV was detected only in *A. agrarius* and SEOV only in *R. norvegicus*. The observation that *Hantaan virus*-positive *Apodemus agrarius* (Group A) exhibited the highest number of unique genera suggests a potential association between HTNV infection and alterations in the genus-level composition of this species’ lung microbiota. In contrast, the greater overlap of genera between SEOV-positive (Group B) and SEOV-negative (Group D) *Rattus norvegicus* indicates that the *Seoul virus* may exert a comparatively limited impact on the shared taxonomic composition of the lung microbiota at the genus level in this host species.

**Figure 2:**
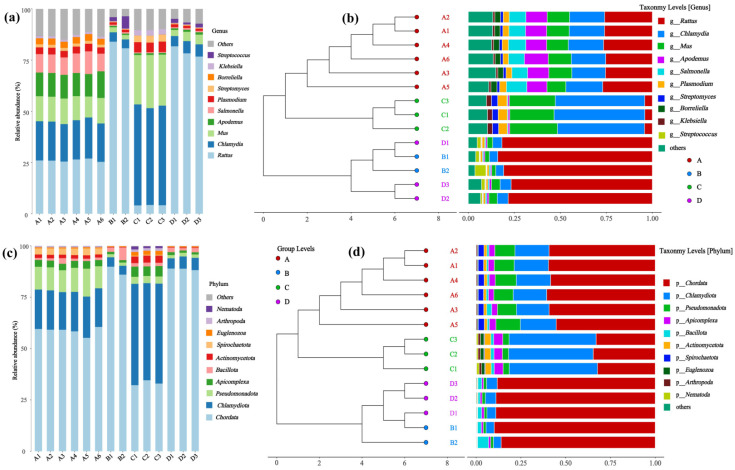
Microbial community composition and clustering in rodent lung tissue samples. (**a**,**b**) Genus- and (**c**,**d**) phylum-level analyses show relative abundance and cluster patterns. Sample sizes per group were as follows: A (n = 6), B (n = 2), C (n = 3), and D (n = 3).

The microbial diversity, abundance, and clustering patterns of different rodent lung tissue samples at the phylum and genus levels are presented in Figure 2. At the genus level (Figure 2a,b), significant differences in relative abundance were observed between genera such as *Streptococcus*, *Klebsiella*, and *Borrelia* in samples from *Hantaan virus*-positive (A) and *Hantaan virus*-negative (C) *Apodemus agrarius*, as well as *Seoul virus*-positive (B) and *Seoul virus*-negative (D) *Rattus norvegicus*. Clustering analysis revealed that Groups A–D each formed distinct clusters, with genera such as *R. Chlamydia* and *R. Salmonella* exhibiting prominent differences in abundance between the groups. At the phylum level (Figure 2c,d), differences in the relative abundance were noted between dominant phyla, including *Chlamydiota*, *Pseudomonadota*, and *Bacillota*, across groups. *Chlamydiota* accounted for a significant proportion of some samples in Group A, while *Pseudomonadota* showed obvious changes in the proportion of some samples in Group C. In the phylum-level clustering analysis, Groups A–D each formed separate clusters, with clear differences in the abundances of phyla such as *Chlamydiota* and *Pseudomonadota* between the groups. In contrast, the diversity differences at the genus level were more refined than those at the phylum level, indicating that the effects of the *Hantavirus* infection (*Hantaan virus* and *Seoul viruses*) and host rodent species (*Apodemus agrarius* and *Rattus norvegicus*) on microbial communities are more specific at the genus level. Intra-group differences between A and C and between B and D reflect the impact of viral infection on lung microbes in the same rodent species. However, inter-group differences between A/C and B/D result from the combined effects of the host rodent species and virus types.

### 3.2. Analysis of Microbial Diversity in Lung Tissues

#### 3.2.1. α-Diversity

**Figure 3:**
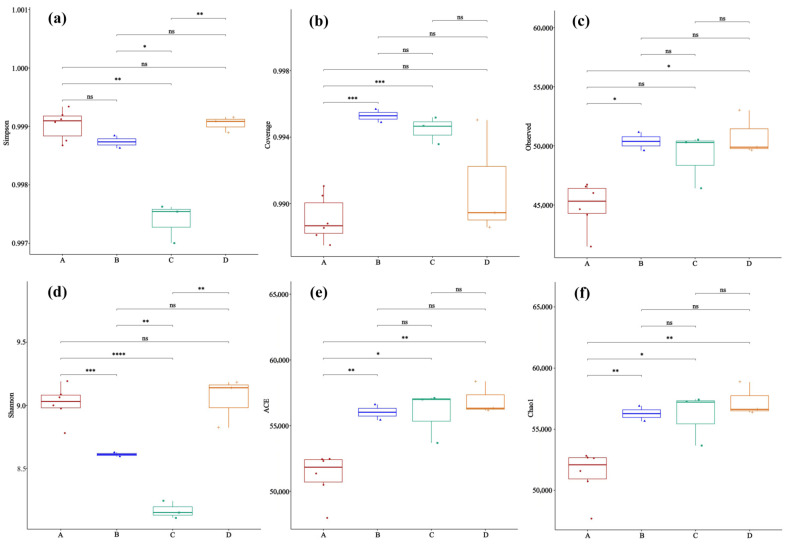
Comparison of the microbial α-diversity between rodent lung sample groups to assess the effects of treatment on the community richness and evenness (ANOVA). (**a**) Simpson index; (**b**) Coverage; (**c**) Observed species number; (**d**) Shannon index; (**e**) ACE; (**f**) Chao1 index. Sample sizes per group were as follows: A (n = 6), B (n = 2), C (n = 3), and D (n = 3). Significance levels: ns, *p* > 0.05; *, *p* < 0.05; **, *p* < 0.01; ***, *p* < 0.001; ****, *p* < 0.0001.

Analysis of the lung microbiota α-diversity revealed that the *Hantavirus* infection and host species background collectively shaped the patterns of the lung microbial community (Figure 3). For the Simpson index (Figure 3a), the index value of *Apodemus agrarius* infected with HTNV (Group A) was significantly higher than that of the uninfected control group (Group C). In contrast, *Rattus norvegicus* infected with SEOV (Group B) and the uninfected group (Group D) were at intermediate levels, with Group B slightly higher than Group D. This pattern is consistent with HTNV infection being associated with an evenness increase in the lung microbial community in *Apodemus agrarius*. However, in the Observed index (Figure 3c), which reflects species richness, as well as the ACE (Figure 3e) and Chao1 indices (Figure 3f), which are used to estimate the total number of species in the community, Group A showed consistently lower values than Groups B-D, as well as a distinct pattern of increased evenness alongside decreased richness. In contrast, after *Rattus norvegicus* was infected with SEOV (Group B), its richness indices showed no significant difference from the uninfected group (Group D) or even a slight increase, suggesting that the host–virus combination is a key determinant of the microbial community response. Additionally, the Coverage index (Figure 3b) for Group A was significantly higher than that of the other groups, indicating more adequate sequencing coverage of the sample community, and the observed decrease in richness was not caused by insufficient sequencing depth. Collectively, these findings highlight differential effects of *Hantavirus* infection on lung microbiota α-diversity depending on the host species, though further studies with larger sample sizes are needed to confirm and generalize these patterns.

#### 3.2.2. β-Diversity

**Figure 4:**
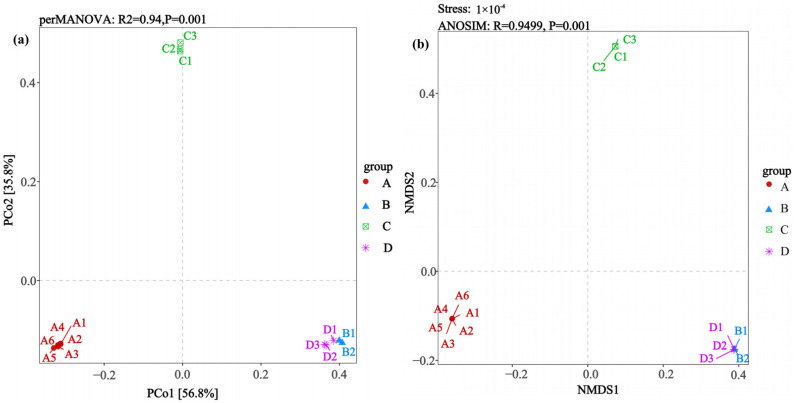
Comparative analysis of β-diversity patterns (via PCoA and NMDS) addressing differences in lung microbiota between *Hantavirus*-infected and *Hantavirus*-uninfected rodent groups. (**a**) PCoA; (**b**) NMDS. Sample sizes per group were as follows: A (n = 6), B (n = 2), C (n = 3), and D (n = 3).

The β-diversity analysis of different rodent lung tissue samples revealed community differences that were associated with the rodent species and *Hantavirus* infection status (Figure 4). The PCoA (Figure 4a) showed that PCo1 and PCo2 explained 56.8% and 35.8% of the community variation, respectively, with a cumulative contribution rate of 92.6%. Statistical validation via perMANOVA indicated extremely significant differences between groups (R^2^ = 0.94, *p* = 0.001). The specific spatial distribution showed that the samples in Group A (*Apodemus agrarius* infected with HTNV) clustered independently on the left side of the coordinate axis. The samples in Group B (*Rattus norvegicus* infected with SEOV) and Group D (uninfected *Rattus norvegicus*) clustered closely on the right side, forming an overlapping region, while the samples in Group C (uninfected *Apodemus agrarius*) distributed independently above the coordinate axis, resulting in the observed spatial separation in the ordination plot. The non-metric multidimensional scaling (NMDS) analysis (Figure 4b) further confirmed this conclusion. Its extremely low stress value indicated excellent dimensionality reduction fitting. The ANOSIM test reconfirmed that the differences between the groups were significantly greater than those within the groups and the sample clustering pattern was highly consistent with that of the PCoA. Group A formed an independent cluster, Groups B/D formed a mixed cluster, and Group C maintained obvious spatial isolation. These multidimensional community distribution characteristics suggest that the rodent species (*Apodemus agrarius* vs. *Rattus norvegicus*) and their *Hantavirus* infection status (HTNV positive/negative vs. SEOV positive/negative) collectively shape the microbial community structure. In summary, Group A (HTNV-infected *Apodemus agrarius*) and Group C (uninfected *Apodemus agrarius*) exhibited the greatest degrees of separation from the other groups.

### 3.3. Comprehensive Effects of Host Species and Hantavirus Infection on the Rodent Lung Microbial Community Structure

**Figure 5:**
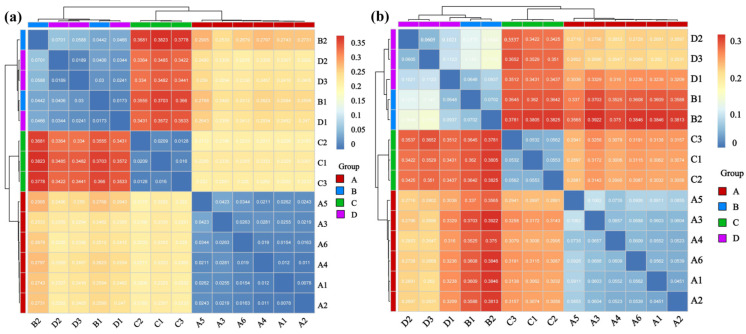
Integrated clustering and correlation analysis revealed alterations in lung microbial communities associated with *Hantavirus* infection. (**a**) Phylum; (**b**) Genus. Sample sizes per group were as follows: A (n = 6), B (n = 2), C (n = 3), and D (n = 3).

Heatmap and cluster tree analyses further revealed the similarities and differences in the microbial community structures between different rodent lung tissue samples (Figure 5). Samples within each group showed high similarity in community structure. The correlation coefficients between samples A1–A6 in Group A (*Apodemus agrarius* infected with HTNV) were generally higher than 0.8, and samples C1-C3 in Group C (*Apodemus agrarius* without infection), samples B1–B2 in Group B (*Rattus norvegicus* infected with SEOV), and samples D1–D3 in Group D (*Rattus norvegicus* without infection) also showed similar high intra-group clustering.

In contrast, comparisons between the groups showed a significant differentiation pattern. The correlation values between Groups A and C (same rodent species with different infection statuses) were mostly distributed in the low range of 0.2–0.4 with distinct color contrast in the heatmap, and the inter-group correlations between Groups A and B/D (different rodent species with different viral infections), as well as between Groups C and B/D (different rodent species with different infection statuses), also remained at low levels (0.3–0.5). The cluster tree further confirmed this trend of inter-group separation with Groups A and C each forming independent clades, and Groups B and D clustering into the same major clade but remaining distinguishable. This pattern is consistent with associations between the rodent species, *Hantavirus* infection status, and differences in microbial community structure.

### 3.4. Host-Specific Remodeling of Lung Microbial Communities and Identification of Differentially Abundant Taxa in Hantavirus Infection

**Figure 6:**
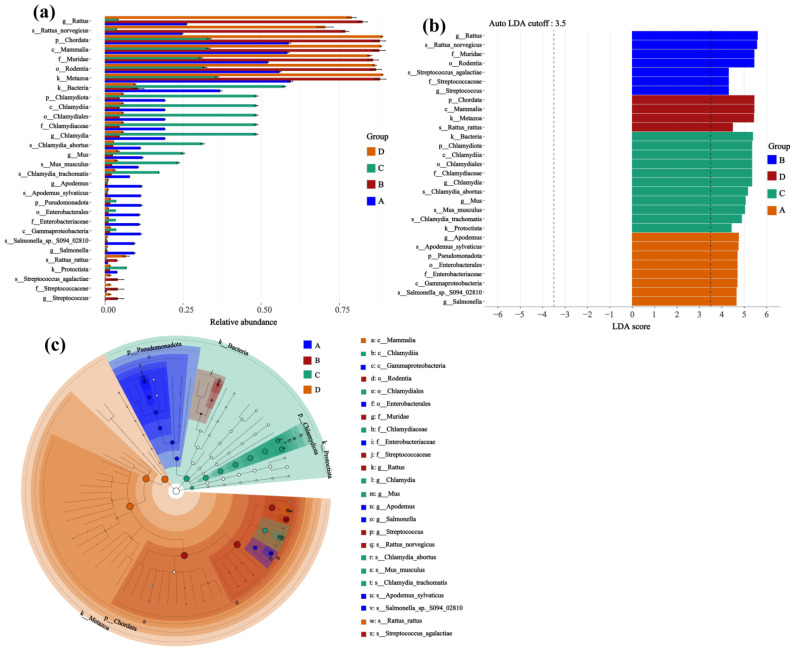
Screening for differentially abundant taxa based on microbial community composition and linear discriminant analysis effect size. (**a**) Relative abundance; (**b**) LDA score; (**c**) Cladogram. Sample sizes per group were as follows: A (n = 6), B (n = 2), C (n = 3), and D (n = 3).

Through microbial composition analysis, effect size assessment, and phylogenetic tree analysis, it was further revealed that the host species and *Hantavirus* infection status co-shaped the specific structure of lung microbial communities (Figure 6). The relative abundance plots (Figure 6a) show that microbial communities in different groups exhibited distinct differential distributions at the phylum and genus levels. Specifically, in the microbial community of Group C (uninfected *Apodemus agrarius*), the genus *Chlamydia* (phylum *Chlamydiae*) and the species *Chlamydia abortus* were more abundant. In contrast, Group B (*Rattus norvegicus* infected with SEOV) showed a higher representation of taxa within the family *Streptococcaceae*; for example, *Streptococcus* and *Streptococcus agalactiae* were detected with higher abundances. Meanwhile, Group A (*Apodemus agrarius* infected with HTNV) and Group D (uninfected *Rattus norvegicus*) each had their own unique characteristic microbial taxa, indicating significant differences in species abundance between the groups.

These group-dependent differentiations in microbial community structure were statistically supported by the LDA (Figure 6b). This analysis indicated that *Streptococcus agalactiae* and Streptococcus were identified as potential discriminant features for Group B (infected Rattus norvegicus) with high LDA scores, while *Chlamydia* and *Chlamydia abortus* showed prominence as characteristic taxa in Group C (uninfected *Apodemus agrarius*), suggesting they were relatively more abundant in this group. Furthermore, the taxonomic tree (Figure 6c) visually confirmed from a phylogenetic perspective that phylum-level taxa such as *Pseudomonadota* and *Chlamydiota* exhibited unbalanced distributions across Groups A–D. This observation reveals that differences in rodent species and viral infection status are associated with variations in the lung microbiota taxonomic composition.

## 4. Discussion

### 4.1. The Interplay Between Hantavirus and the Lung Microbiota in Viral Transmission

This study provides preliminary, hypothesis-generating insights into the associations between *Hantavirus* infection, host species, and lung microbiota. A key constraint is the small and uneven sample size, which limits the statistical power and generalizability. Therefore, the observed patterns require validation in larger, balanced cohorts. *Hantavirus* infection and the host rodent species collectively shape the lung microbial community structure [17]. Our findings that the lung microbiota structure is associated with the host species and *Hantavirus* infection status invite speculation into potential mechanisms linking microbial shifts to viral ecology. The identified taxa are of particular interest due to their known associations with infectious disease processes in animal hosts. *Klebsiella*, an opportunistic pathogen, can cause pneumonia and sepsis in species such as mink and fox, with its toxins and biofilms potentially compromising host immune barriers [18]. In rodents, analogous mechanisms might alter immune responses and thereby affect viral transmission. *Chlamydia*-related members are recognized for their ability to infect respiratory tracts and induce persistent inflammatory states [19], while *Hantavirus* infection itself involves significant immunopathology [20]. It is therefore plausible that changes in these microbes’ abundances are linked to alterations in the immune status and local virological niche of the host. Furthermore, some members of the *Pseudomonadota* phylum can influence the systemic host metabolism and immunity, potentially participating in a broader interaction network between the virus, microbiota, and host that may indirectly affect the viral transmission efficiency [21].

Interpretation of specific low-abundance taxa warrants caution due to methodological limitations common in low-biomass studies. While qPCR on extraction blanks indicated that the amount of reagent contamination was below detection levels, the absence of sequenced negative controls limits the ability to bioinformatically identify and subtract potential contaminant sequences. Therefore, while the group-level diversity patterns are robust, inferences about specific taxa, especially those identified as differentially abundant, should be considered exploratory. Consequently, taxa highlighted by analyses such as LEfSe (such as *Streptococcus* and *Chlamydia*) are strictly hypothesis-generating. Their proposed association with infection status and any implied functional role in virus–host interaction remain speculative and require validation in independent, larger studies that incorporate rigorous contamination controls.

### 4.2. Host-Specific Changes in Microbial α-Diversity Following Hantavirus Infection

The changes in community evenness and richness after infection observed in this study are consistent with the theory that viruses remodel the host microenvironment through immune responses [22]. *Hantavirus* infection is known to elicit a strong immune-inflammatory response in the host. As the target organ, lung tissue undergoes significant alterations in its cytokine environment [17]. However, the specific mechanistic pathways leading to the observed microbial shifts, such as higher Simpson index/evenness but lower richness in HTNV-infected *A. agrarius* (Group A) and the similarity between SEOV-infected *R. norvegicus* (Group B) and uninfected *R. norvegicus* (Group D), were not directly measured in this study. We therefore propose the following hypothesis for future validation: One possible mechanism involves established *Hantavirus* immune evasion strategies, such as inhibiting interferon responses via Gn-TRAF3 interaction and blocking NF-κB nuclear translocation via the nucleocapsid protein hijacking importin-α [23,24]. Such immune regulation could alter the pulmonary microenvironment, potentially inhibiting the growth of some sensitive microbial taxa (reducing richness) while providing new niches for opportunistic pathogens (affecting evenness). These alterations may lead to functional consequences, including the loss of protective commensals that contribute to host defense and tissue homeostasis.

This interpretation aligns with the concept of virus–host co-evolution. Inherent differences in the genetics, physiology, and basal immunity of *A. agrarius* and *R. norvegicus* likely shape their foundational microbiomes [25], and consequently, their microbial response to viral stress [26]. The pronounced shift in *A. agrarius* upon HTNV infection versus the stability in *R. norvegicus* upon SEOV infection may mirror differing stages or strategies in host–virus adaptation. In nature, biodiversity such as complex rodent community structure is negatively correlated with the risk of *Hantavirus* transmission [27]. This study extends this pattern to the microbial realm at the microscale. Viral infection may reduce pulmonary microbial diversity and impair ecological function for host health and stability. However, small sample sizes, particularly in control groups (n = 3), render these patterns and hypotheses preliminary. Validation in larger, controlled experiments is necessary. Additionally, confounding of host species and virus type in field samples prevents full distinction of their effects or interaction. While intriguing, these host-specific patterns are preliminary due to sample size limitations and the confounding of the host species with the virus type in our design. Future controlled experiments are needed to disentangle these effects.

### 4.3. Host Genetics and Viral Infection Jointly Drive Lung Microbial β-Diversity

The strong β-diversity separation driven by the host species and, to a variable extent, infection status aligns with known determinants of microbiome assembly. Host genetics can shape mucosal environments, thereby selecting for distinct microbial communities [28,29], which likely explains the baseline differences between uninfected *A. agrarius* and *R. norvegicus* (Groups C vs. D). Viral infection may then layer additional selective pressures. *Hantavirus* perturbs host cell signaling and immune pathways [23,24], potentially altering the metabolic and immune landscape of the lung to create new niches or eliminate existing ones. The patterns of β-diversity differentiation may be closely associated with changes in the metabolic microenvironment. Viral infection induces tissue hypoxia and energy metabolism reprogramming [30]. Recent studies have shown that intestinal microbial products can indirectly affect pathogen colonization by maintaining the epithelial barrier integrity. Furthermore, the imbalance of such metabolic interactions may be a key reason for the deviation of community structure in the infected group from the uninfected baseline [31]. Furthermore, the host genetic background (such as species-specific differences in mucus components between rodent species) and virus-induced inflammatory environments jointly influence new microbial niches [32,33]. Among these, microbiota with specific metabolic capabilities are suggested to be enriched in hosts with specific genetic backgrounds. This phenomenon suggests that viral modifications may indirectly select for microbial taxa with specific metabolic characteristics. Such selection may occur by affecting glycosylation patterns on host mucosal surfaces or nutrient availability. This could ultimately contribute to the distinctness of communities in Group A from those in uninfected conspecifics (Group C). Additionally, these communities are significantly different from those derived from *Rattus norvegicus* (Groups B/D) [34].

Such changes in lung microbial community structure are potentially co-shaped by host genetics and viral infection. These changes may regulate local immune homeostasis or affect epithelial barrier function. Thereby, they represent a potential mechanism influencing the host adaptation of *Hantavirus* and their persistence in natural hosts. Notably, the relative proximity between Group B (*Rattus norvegicus* infected with SEOV) and Group D (uninfected *Rattus norvegicus*) in β-diversity analysis suggests that SEOV exerts a relatively mild effect on the lung microbial community. This may reflect a more synergistic evolutionary relationship between the virus and its primary host. In contrast, the more pronounced impact of HTNV on the lung microbiota of *Apodemus agrarius* (its primary host) may represent another mode of interaction. Given the exploratory nature and limited sample size of this study, these interpretations regarding the differential effects of HTNV and SEOV should be viewed with caution. While consistent with the data, they are considered hypothesis-generating for future validation in larger, statistically powered cohorts.

### 4.4. Hantavirus–Host Coevolution Shapes Microbial Community and Influences Viral Transmission

Community clustering patterns, primarily separated by the distinct host–virus pairings, are highly consistent with the theoretical framework of *Hantavirus*–host coevolution [28]. Phylogenetic studies have confirmed long-term coevolutionary relationships between Hantavirus and their specific host animals [35]; for example, HTNV-like viruses are primarily associated with *Apodemus agrarius*, while SEOV-like viruses correspond to *Rattus norvegicus* [36,37]. This virus–host specific association likely determines the baseline composition and stress response patterns of downstream microbial communities, and is further supported by epidemiological studies indicating that geographic isolation and niche differentiation drive the formation of distinct viral genetic lineages [38]. This corroborates the result in the present study that Group B (*Rattus norvegicus* infected with SEOV) and Group D (uninfected *Rattus norvegicus*) can still be distinguished despite high similarity, indicating that viral infection may introduce additional community selection pressure even within the same rodent species. Moreover, the heterogeneous landscape of our sampling region–comprising mixed agricultural, woodland, and peri-domestic habitats–may further modulate rodent population structure and their microbial exposures. Future studies explicitly documenting habitat variables at the trap-level would help disentangle the relative contributions of environmental, host, and viral factors to lung microbiome assembly.

Such differences in community structure may influence the transmission and pathogenicity of *Hantavirus* through multiple mechanisms. Studies have shown that *Hantavirus* infection can invade vascular endothelial cells by binding its glycoproteins to integrin receptors. This perturbation of the innate immune signaling of host reshapes the pulmonary microenvironment and thereby affects microbial colonization in a differential manner [39]. For example, one hypothesis is that infection of *Apodemus agrarius* with HTNV (Group A) may trigger a stronger local inflammatory response. In contrast, the immune tolerance of *Rattus norvegicus* to SEOV (Group B) may mitigate such changes. This explains why the community differentiation between Groups A and C (same rodent species with different infection statuses) is even greater than that between Groups A and B (different rodent species with different viruses). Furthermore, human-activity-induced land use changes, such as land consolidation and agricultural expansion, may further amplify the impact of such microbial community differences on virus transmission by simplifying the rodent community structure.

### 4.5. The Mediating Role of Microbes in Hantavirus–Host Interactions

Potential associations were observed between key differential microbial taxa and infection by specific *Hantaviruses* within their respective natural hosts, warranting exploratory consideration of their possible roles. Notably, the genera *Chlamydia* and *Streptococcus* were among the taxa that showed differential abundance across groups in our analysis. *Chlamydia* species are known as respiratory pathogens capable of modulating the host immune microenvironment, while *Streptococcus agalactiae* is an opportunistic pathogen involved in microbial community interactions and host immune modulation. These general properties are, at a broad level, consistent with the types of immune alterations associated with *Hantavirus* infection [40]. Based on these general parallels, it is plausible to speculate that host-specific immune responses to infection might create niches for microbes with immunomodulatory potential. If present and active, such microbes could, in theory, influence the local immune milieu and thereby potentially affect the microenvironment for viral replication [18].

At the level of microenvironmental and resource competition, *Hantavirus* replication hijacks host cell metabolism. For instance, studies have suggested that the virus may shut down cholesterol and lipid metabolic pathways in lung cells. Such virus-driven metabolic reprogramming, combined with potential infection-induced tissue hypoxia, may collectively alter the lung’s nutrient supply and redox potential [30]. One speculative possibility is that these altered conditions might exert selective pressure on the resident microbial community, potentially favoring groups adapted to the new environment and contributing to community restructuring. This could offer a hypothetical framework to interpret the observed increase or decrease in specific taxa.

However, these functional interpretations regarding *Streptococcus agalactiae* and *Chlamydia abortus* remain highly speculative and must be framed with considerable caution. The current findings are based on a small sample size and, importantly, lack specific sequencing-based contamination controls for these taxa, which limits the strength of biological inference. Their prominence in our LEfSe results should therefore be viewed strictly as preliminary observations generating hypotheses for future validation, rather than as evidence of definitive functional roles in the context of *Hantavirus* infection.

In summary, this exploratory study applied multi-angle microbiome analyses to provide initial evidence that the host species and *Hantavirus* infection status are associated with differences in the lung microbial community structure, highlighting several microbial taxa of interest. These findings offer preliminary clues for understanding the complex interactions between *Hantavirus*, the host, and the lung microbiome. A key study highlight is the identification of specific taxa, such as *Chlamydia* and *Streptococcus*, that may be linked to *Hantavirus* infection. If confirmed, these associations could motivate future experimental research using germ-free animal models or in vitro systems to investigate potential causal mechanisms in *Hantavirus* infection dynamics, ultimately contributing to a theoretical foundation for novel intervention strategies.

However, this study has important limitations. The small sample size and lack of rigorous contamination controls necessitate caution in interpreting the results. Furthermore, the absence of detailed habitat metadata, such as precise land cover types at trapping sites, limits our ability to assess how the immediate environmental context interacts with the infection status to shape the lung microbiome. Future studies should prioritize larger sample sizes, stringent contamination controls, and standardized ecological surveys to capture environmental variables. Longitudinal designs would help clarify the temporal relationships between infection, microbiome changes, and environmental factors, strengthening the validity and applicability of the findings.

## 5. Conclusions

This study provides preliminary evidence that natural host—*Hantavirus* pairings are associated with distinct structures and compositions of the lung microbial community. Specifically, in *Apodemus agrarius*, HTNV infection significantly increases the evenness of the lung microbial community but significantly decreases its species richness. In contrast, no significant reduction in microbial richness was observed in *Rattus norvegicus* following SEOV infection, suggesting potential specificity linked to the host—virus pairing in the microbial response. β-diversity analysis further indicated that lung microbial community composition varied in relation to both rodent species and infection status, with the most pronounced difference observed between the two host species. Analysis of differentially abundant taxa identified specific microbial features across groups. For instance, *Streptococcus* was found to be a discriminating feature in SEOV-infected *Rattus norvegicus*, while *Chlamydia* was a characteristic taxon in uninfected *Apodemus agrarius*. These associations are preliminary and require validation. This exploratory study provides initial, proof-of-concept evidence that *Hantavirus* infection may be associated with changes in the lung microbiome of natural rodent hosts, and that this association appears to be influenced by both host species and virus type. The specific roles of the identified taxa and the mechanisms underlying the observed community shifts remain to be conclusively determined. Future studies with larger sample sizes, controlled experimental designs, and mechanistic models are required to validate these associations and establish causality.

## Figures and Tables

**Table 1:** The qPCR results of different samples.

Samples	Ct Values (16S rRNA)	Ct Values (ITS)
A	31	-
B	33	-
C	31	42
D	35	-
E	-	-
F	ND	-
G	-	-
H	-	-

A and C represent DNA samples from *Apodemus agrarius* that were *Hantaan virus*-positive and *Hantaan virus*-negative, respectively; B and D represent DNA samples from *Rattus norvegicus* that were *Seoul virus*-positive and *Seoul virus*-negative, respectively; E–H represent DNA samples from blank controls.

## Data Availability

The original contributions presented in this study are included in the article. Further inquiries can be directed to the corresponding author.

## Abbreviations

The following abbreviations are used in this manuscript:

LEfSe	linear Discriminant Analysis Effect Size
HTNV	*Hantaan virus*
ACE	Accumulated cyclone energy
LDA	Linear Discriminant Analysis
OTUs	Operational Taxonomic Units
